# Sex Differences in Children with Uncomplicated Attention Deficit/Hyperactivity Disorder and Sleep Problems

**DOI:** 10.3390/children11060636

**Published:** 2024-05-24

**Authors:** Annelie Lindholm, Håkan Jarbin, Katarina Aili, Jens M. Nygren, Petra Svedberg, Ingrid Larsson

**Affiliations:** 1 Department of Health and Care, School of Health and Welfare, Halmstad University, SE-30118 Halmstad, Sweden; annelie.lindholm@hh.se (A.L.); jens.nygren@hh.se (J.M.N.); petra.svedberg@hh.se (P.S.); 2 Child and Adolescent Psychiatry, Department of Clinical Sciences Lund, Lund University, SE-22184 Lund, Sweden; hakan.jarbin@regionhalland.se; 3 Child and Adolescent Psychiatry, Region Halland, SE-30185 Halmstad, Sweden; 4 Department of Health and Sport, School of Health and Welfare, Halmstad University, SE-30118 Halmstad, Sweden; katarina.aili@hh.se

**Keywords:** attention deficit/hyperactivity disorder, ADHD, paediatrics, sleep problems in children

## Abstract

Background: Approximately 7.6% of children are diagnosed with attention deficit/hyperactivity disorder (ADHD), and sleep impairments affect 25–85%. There is a noticeable lack of research on girls and sex differences. The aim of this study was to examine sex differences in children with uncomplicated ADHD and sleep problems. Methods: Cross-sectional baseline data were retrieved from a randomized controlled trial with weighted blankets (55 boys and 41 girls, 6–14 years) on a cohort recently diagnosed with uncomplicated ADHD and sleep problems. Differences between boys and girls in ADHD symptoms, objectively and subjectively measured sleep, anxiety, and functioning were examined via parent- or self-reported validated instruments. Results: Girls reported significantly lower (worse) satisfaction with well-being, life overall, and school, but not for family. Parents reported more sleep anxiety and night-time wakings among boys, but no sex differences in other measures and also not in self-reported measures or objective sleep measures. Children who reported worry, sadness, or unhappiness had more sleep problems. Conclusions: Boys with ADHD and sleep problems may need support with sleep-related anxiety and night-time wakings, while girls may require support with overall functioning. Additionally, children who express feelings of worry, sadness, or unhappiness alongside their ADHD symptoms should have attention given to their sleep.

## 1. Introduction

Attention deficit/hyperactivity disorder (ADHD) is among the most frequent psychiatric disorders in children, with prevalence rates above 7.6% worldwide [1]. ADHD is characterised by hyperactivity, inattention, and impulsivity, and the incidence of these symptoms is heterogenous [2]. In addition to a genetic predisposition, it has been shown that the aetiology also appears to be influenced by socioeconomic situation [3]. More than 50% of diagnosed children have sustained symptoms as adults [4]. About twice as many boys than girls are diagnosed [5]. Swedish national data for children aged 10–14 report a strong over-representation of boys, albeit approaching the expected ratio from epidemiological data (male preponderance in 2006 was 4.1:1, 3.3:1 in 2013, 2.7:1 in 2016, and 2.2:1 in 2021) [6]. Historically, more studies on boys have been made, which has given rise to challenges in identifying and treating girls [7], underscoring the necessity for additional research studies focused on girls [8,9,10]. Improved understanding of the behavioural characteristics of ADHD presentation in girls is needed in order to improve assessment and treatment [11]. Previous research has revealed that girls with ADHD less frequently exhibit symptoms of impulsivity and hyperactivity but tend to suffer from inattention more often than boys [12].

ADHD is commonly associated with impaired sleep, and both the children and their parents report such complaints [13]. It is unclear whether ADHD causes sleep problems per se, or if sleep problems exacerbate ADHD symptoms [14]. Either way, sleep problems may lead to a worsening of ADHD-related symptoms, as they affect cognitive functioning [15,16] and have been associated with worse physical functioning in children with ADHD [17]. Furthermore, sleep problems in children with ADHD often lead to more issues for the child [18,19,20] and the family [14,18,21], such as a worsening of daily functioning, ADHD symptom severity [14], and quality of life [20]. Sufficient sleep is associated with better mental health in children [22], both in long- and short-term perspectives [23], while insufficient sleep is associated with negative effects on both mental and physical health [22]. Earlier studies have reported sleep-related problems in 25–85% of children with ADHD [20,24,25,26,27,28], compared to 20–30% in non-diagnosed children [29]. Research has demonstrated that girls with ADHD experience worse sleep issues than boys with ADHD [30]. Additionally, previous studies have indicated that girls with ADHD are more likely to report sleep difficulties than boys, although these studies have been limited in number, underscoring the need for further research [30]. Children with ADHD frequently reported sleep problems compared to other children of the same age, including bedtime refusal, trouble initiating sleep, difficulty staying asleep, daytime sleepiness, and fractured sleep [31]. However, there is an inconsistency in the types of sleep problems compared between studies using objective measures like actigraphy and polysomnography (PSG) versus those using subjective measurements such as self- or parent-reported questionnaires [14]. Studies examining children with ADHD through objective measures have sometimes reported contradictory results, such as variations in total sleep time (TST) [14,32,33]; for instance, both longer [32] and shorter TSTs measured by using PSG [33] have been described. In contrast, studies using subjective measures have generally demonstrated consistency in reported sleep-related problems, where the following are the most frequently reported [14]: difficulties falling asleep [34,35], snoring [35,36], parasomnias [37,38], nightmares, [34], short TST [39], and daytime sleepiness [34,37]. More studies regarding both objectively and subjectively measured sleep in children with ADHD are needed in order to gain a better understanding of the area and to support a better quality of life in this group. Regarding normal sleep habits, sleep requirements vary by age; therefore, recommendations differ for different age groups. Children aged 6–12 years are recommended to get 9–12 h of sleep across a 24 h period [40].

It is important to correctly measure sleep quality and other sleep-related factors [41]. As mentioned above, sleep can be measured by both objective and subjective measures. The term ‘sleep quality’ often refers to several sleep measures: sleep onset latency (SOL), TST, wake after sleep onset (WASO), and sleep efficiency (SE). One commonly used objective measure is ActiGraph, which bases its information on activity levels. Videosomnography and polysomnography are other commonly used objective tools, but these are both relatively expensive and time-consuming [41]. Sleep questionnaires are an example of a subjective measurement, targeting factors such as sleep habits, sleep patterns, breathing difficulties, sleepiness during daytime, and parasomnias [41]. Sleep questionnaires can be reported by parents or self-reported. It has been shown that reports from children as young as eight-years-old can be reliable and valid when age-appropriate questionnaires are used [42]. Sleep problems in children without ADHD have been associated with a lower quality of life and symptoms such as anxiety and depression [43]. However, there is a lack of similar studies involving children with ADHD. Therefore, it is of interest to investigate whether children with ADHD and sleep problems who report feeling worried, sad, or unhappy exhibit more pronounced sleep problems, ADHD symptoms, and impaired satisfaction with functioning in daily life than children without these symptoms.

The primary aim of this study was to compare boys versus girls with uncomplicated ADHD and sleep problems (diagnosed by a senior child and adolescent psychiatrist when attending an ADHD unit and according to DSM-5) regarding their ADHD diagnosis subtype, objectively measured sleep, parent- and child-reported sleep issues, parent-reported ADHD and anxiety symptoms, and self-reported satisfaction with functioning in daily life. We hypothesised that boys with ADHD and sleep problems would more often suffer from combined or hyperactive ADHD, more sleep-related problems, more ADHD and anxiety symptoms, and worse satisfaction with functioning in daily life than girls.

A secondary aim was to compare children who reported being sad, worried, or unhappy compared to children not reporting these problems regarding ADHD diagnosis subtype, objectively measured sleep, parent- and self-reported sleep issues, ADHD and anxiety symptoms, and satisfaction with functioning in daily life. We hypothesised that children who reported problems would have more sleep issues, ADHD and anxiety symptoms, and worse satisfaction with functioning in daily life.

## 2. Materials and Methods

### 2.1. Study Participants and Design

This study is cross-sectional, with baseline data from a cohort of children participating in a randomised controlled study [44]. This study included 96 children—55 boys and 41 girls aged 6–14 years. They were all recently diagnosed with uncomplicated ADHD, had sleep problems, and had consented to participate in a sleep intervention with weighted blankets. Uncomplicated ADHD was defined as ADHD without significant comorbidity or social burden. The sample was homogenous, with an over-representation of white children, similar to the overall population in Sweden. The study protocol, recruitment process, and sample representativeness are reported in detail elsewhere [45]. The participants and their parents were recruited between January 2020 and January 2022 when attending the ADHD unit at a Child and Adolescent Mental Health Service (CAMHS) in the south of Sweden. This initiative was created to diminish waiting lists and delays from referral to treatment [46]. Children were triaged to this unit when the structured Brief Child and Family Phone Interview indicated a probable diagnosis of uncomplicated ADHD, encompassing about half of established ADHD diagnoses. The assessment at the unit was based on written information from the child’s school, including open questions regarding behaviour, strengths, and difficulties with learning and social functioning at school, a teacher ADHD rating scale, observation of the child, and a thorough diagnostic interview with the child and parent(s). The interview was performed by a resident, and a diagnosis was established by a senior child and adolescent psychiatrist at the end of each assessment. Children diagnosed with ADHD were offered parental group psychoeducation and a letter to the child’s school informing them about the diagnosis and any necessary adaptations to the curriculum. In about half of the cases, medication was also prescribed. Children with more complicated ADHD, such as significant comorbidity, intellectual impairment, or living in families with severe parental stress, were not referred to this unit but treated at the regular CAMHS, since they needed a more intense follow-up.

All the children diagnosed with ADHD at the unit were eligible to participate in this study without any exclusions. The research project was reviewed and approved by the Ethical Review Authority in Sweden (Nr: 2019-02158) and was made in accordance with the Code of Ethics of the World Medical Association. The children and parents were informed verbally and in writing about this study and were provided written informed consent.

### 2.2. Measurements

Sex of the child, age of parents, parental education, as well as age, weight, and height of the child were reported by the parents in the baseline questionnaire. Information regarding ADHD diagnosis and medication was collected from medical records.

#### 2.2.1. ActiGraph

ActiGraph is commonly used as an objective measure of sleep [41], and it has been shown to be valid in several studies [47]. In this study, measurements were performed by using a wrist-worn Motionwatch 8 (CamNtech Ltd. Fenstanton, UK). This ActiGraph has a tri-axial accelerometer that uses MEMs technology capable of sensing motions in the resultant force range between 0.01 and 8.00 g. It registers total gross motor activity for further sleep–wake analyses in the software program Actiwatch Activity & Sleep Analysis (CamNtech Ltd., version 7.38). The participants were instructed to wear the Motionwatch 8 on the non-dominant wrist and to push the button when it was time to sleep or when the lights were out. They were also instructed to put it on one to two hours before going to sleep or to wear it 24 h per day, depending on their preference and daily routines. Most participants only wore the watches at night, and only a few children used the watch 24 h a day. Measurements were carried out during seven nights each measurement week only on regular schooldays and weekends, with no measurement during holidays. Total sleep time (TST), sleep onset latency (SOL), wake after sleep onset (WASO), and sleep efficiency (SE) were used in this study.

#### 2.2.2. Children’s Sleep Habits Questionnaire

The Children’s Sleep Habits Questionnaire (CSHQ) is a validated and reliable 45-item questionnaire [48] that has been used in several studies to measure sleep quality [49]. The instrument has also been translated into Swedish and validated in that version by research group members [50]. The parents answered the questionnaires and measured sleep behaviour in children of younger ages. The questionnaire was divided into eight subdomains, with one to eight questions in each subdomain: bedtime behaviour; sleep onset delay; sleep duration; anxiety around sleep; behaviour occurring during sleep and night-time wakings; parasomnias; sleep-disordered breathing; and morning waking/daytime sleepiness. The parent was instructed to base his or her answers on a ‘typical’ recent week, and there were three answering alternatives: 3 (usually, 5–7 times/week), 2 (sometimes, 2–4 times/week), and 1 (never/rarely, 0–1 time/week). In this study, we also used the total CSHQ score.

#### 2.2.3. Insomnia Severity Index

The Insomnia Severity Index (ISI) is a seven-item questionnaire that is reliable [51], validated, and commonly used globally to evaluate insomnia in adolescents [52]. The Swedish version of the questionnaire was used. In this study, the individual questions were simplified to also add clarity for the younger children in our cohort. The questions cover difficulties falling asleep, problems staying asleep, daytime symptoms depending on sleeping problems, and worry regarding sleeping problems. Each item is graded between 0 and 4, where 0 represents no problems, 1 mild problems, 2 moderate problems, 3 severe problems, and 4 very severe problems.

#### 2.2.4. The Parent-Reported Swanson, Nolan and Pelham Rating Scale

The parent-reported Swanson, Nolan and Pelham rating scale (SNAP IV-parent) is widely used in research and clinical practice to assess parent- or teacher-reported symptoms of ADHD and oppositional defiant disorder (ODD). The instrument is a valid and robust measure [53]. The Swedish version of the questionnaire was used. The 18-item scale (SNAP IV-parent) reported by the parents was used to assess the severity of ADHD symptoms [54]. SNAP IV-parent consists of nine questions about attention deficits, six questions regarding hyperactivity, and three questions regarding impulsivity, rated on a four-point Likert-like scale from 0 (not at all) to 3 (very much). The total scores of the nine attention deficit items, items 1–9, as well as a total score of the nine hyperactivity and impulsivity items, items 11–19, were used in this study.

#### 2.2.5. EuroQol-5 Dimensions-Youth

The EuroQol-5 Dimensions-Youth (EQ-5D-Y) was developed from the adult version of the EQ-5D. The instrument has been shown to have good reliability and validity in children [55]. The version translated to Swedish was used in this study [55]. The results were self-reported by the children. The five items on mobility, self-care, usual activities, pain or discomfort, and anxiety, depression, or sadness all had three response alternatives: 1, no problems; 2, some problems; and 3, a lot of problems. The included visual analogue scale (VAS) represents a subjective health rating regarding the current overall health status, shown as a scale from 0 (the worst health state the child can imagine) to 100 (the best health state the child can imagine).

#### 2.2.6. Child Outcome Rating Scale

The five-item child outcome rating scale (CORS) is a questionnaire that aims to measure psychological distress in youths and young children [56]. The CORS was developed for children between 6 and 12 years and contains child-friendly language, smiley faces, and frowny faces [56]. This instrument has good validity and moderate reliability [57]. The Swedish version of the instrument was used. This scale measures how well a child is satisfied with individual well-being, interpersonal relationships (mostly family), school, and life overall. The children were instructed to mark their answers on a 10 centimetre line beginning with a frowning face and ending with a smiling face, scoring from 0 (lowest/worst) to 10 (highest/best).

#### 2.2.7. The Short Form of the State-Trait Anxiety Inventory

The short State-Trait Anxiety Inventory (STAI) is a shorter form of the STAI that is used when the full form is unnecessary to evaluate anxiety [58]. This instrument has good reliability and validity in children [59]. The Swedish version of the instrument was used. In this study, the short form was used and self-reported by the children, who were asked to respond to six statements by indicating how they felt that each statement applied to them. The State-Anxiety item includes questions regarding topics such as: “I am tense; I am worried; I feel calm; I feel secure”. The Trait anxiety item includes statements like: “I worry too much over something that really does not matter”; “I am content”; “I am a steady person”. The items are rated on a four-grade scale (1–4), where 1 means almost never and 4 means almost always. Higher scores indicate greater anxiety in a child.

### 2.3. Statistics

Both parametric and non-parametric tests were used since some variables were normally distributed while others lacked a normal distribution. For non-normally distributed variables, median values and, with some exceptions, mean and standard deviation values were also reported. Regarding the characteristics of the sample, a Mann–Whitney U test was used to analyse median values and to compare ages between sexes. Chi-Squared tests or Fisher’s exact tests (depending on the number of participants in each group) were used to compare diagnoses between the sexes, the proportion of children in the different IsoBMI groups, parental educational background, and parental age groups. When analysing ActiGraph results, the CSHQ-subdomains, and total scores (except the subdomain daytime sleepiness), as well as total ISI and short STAI scores, Mann–Whitney U tests were used to analyse differences between the sexes. Regarding the CSHQ subdomain of daytime sleepiness as well as total scores in inattention and hyperactivity in the SNAP-IV-parent, Student’s *t*-tests were used.

In all the EQ-5D-Y items except mobility and the EQ-5D-VAS, Fisher’s exact tests were used to analyse the number of responses in each group as well as differences between the sexes. A Chi-squared test was used for the mobility item. In the EQ-5D-VAS, a Mann–Whitney U test was used. For differences between the sexes regarding CORS total score and the individual variables, Mann–Whitney U tests were used to analyse differences between the sexes.

Due to a possible effect of medication on the analysed variables, we tested the variables that showed statistically significant differences between boys and girls by separately analysing medicated and unmedicated children.

When dividing the children according to those who reported no problems regarding the EQ-5D-Y item of feeling worried, sad, or unhappy and those who reported some/a lot of problems, Mann–Whitney U tests were used to analyse differences between the sexes regarding CSHQ, ISI, short STAI, and CORS. SPSS (v.25.0; IBM Corp., Armonk, NY, USA) was used in all the statistical analyses. A *p*-value < 0.05 was considered to be statistically significant.

## 3. Results

### 3.1. Demographic Characteristics

**Figure 1:**
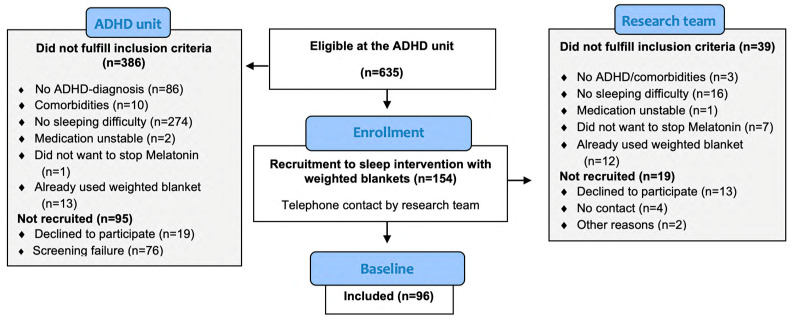
CONSORT flow diagram of the recruitment and patients enrolled in this study.

A total of 96 children, 57% (55) boys and 43% (41) girls, aged 6–14 years were included. The girls were, on average, older than the boys: 10 versus 9 years (*p* = 0.011) (Figure 1, Table 1). Regarding diagnostic subgroups, 26% were diagnosed with attention deficit, 3% with hyperactivity, and 71% with a combination of attention deficit and hyperactivity. The attention deficit ADHD subtype was more common among girls, at 37% versus 18% (*p* = 0.023). Of all the children in the sample, 14 (15%) had comorbidities that did not require primary clinical attention, and oppositional defiant disorder was the most common (7 boys and 1 girl). The other comorbidities were vocabulary disturbance (1 girl), generalized anxiety disorder (1 girl), tics (1 boy and 1 girl), phonologic and grammatic speech disturbance (1 boy), and autism (1 boy). Regarding medication, stimulants were used by 49% of the children, and melatonin by 9%. There were no sex differences regarding the use of melatonin, use of stimulants, overweight, or obesity. Most parents were between 31 and 40 years old and had attended university. Only very few had elementary school as their highest education.

### 3.2. Objectively Measured Sleep

When measured using ActiGraph, there were no statistically significant differences among the sexes regarding the four parameters: TST, SOL, SE, or WASO (Table 2).

### 3.3. Subjectively Measured Sleep

In Table 3, comparisons between boys and girls regarding parent-reported CSHQ- and child-reported ISI results are shown. There were statistically significant differences between girls and boys regarding sleep anxiety and night-time wakings, wherein the boys had higher values. Parents reported that both boys and girls had values above the cut-off point for clinical sleep disturbances. Three boys and three girls had values below the cut-off value. Children reported values below the level of clinical insomnia with no difference between the sexes.

### 3.4. ADHD and Anxiety Symptoms

Regarding SNAP IV-parent, the results showed that parents of both sexes reported that their children had mild-to-moderate or severe symptoms. Significant differences were seen between boys and girls regarding inattention, wherein the parents of girls reported higher values (*p* = 0.039) (Table 4). Regarding hyperactivity/impulsivity, there were no statistically significant differences between the sexes (*p* = 0.318). Boys and girls reported the same level of anxiety (short STAI).

### 3.5. Functioning in Daily Life

Regarding the EQ-5D-Y items, all boys and nearly all girls chose the response alter-native “no problems” in mobility: 100% and 95% in boys and girls, respectively (Table 5). Regarding self-care, the percentages of children reporting no problems were 64% in boys and 73% in girls, respectively. The children rated the remaining items lower, and the “no problem” alternative ranged between from 49% to 80% in the groups, while hardly any of the children chose the response option “a lot of problems” for mobility. Regarding the current health state, the groups rated their health near the top of the scale. The results showed no significant differences between the sexes regarding any of the EQ-5D-Y items. Girls reported less satisfaction with functioning, well-being, school, and life overall but not with family.

To examine the possible effect of medication, variables that showed statistically significant differences between boys and girls, including diagnosis, parasomnias and night-time awakenings (CSHQ); inattention (SNAP IV); and well-being, school, and life overall (CORS) were analysed further. In these analyses, children with medication were compared to those without. No statistically significant differences were found, with *p*-values ranging from 0.107 to 0.926.

### 3.6. Differences between Children Who Reported No or Some Problems Such as Feeling Worried, Sad, or Unhappy in EQ-5D-Y

Children reporting problems with feeling worried, sad, or unhappy also reported more sleep issues (ISI), more anxiety (short STAI), lower satisfaction with functioning (CORS), and their parents reported more sleep problems (CSHQ), as seen in Table 6. The other variables examined in these analyses showed no statistically significant differences (see Appendix A).

## 4. Discussion

In our consecutive clinical sample of children with uncomplicated ADHD and sleeping problems, we observed several significant findings: girls who were more frequently diagnosed with the attention deficit subtype expressed lower satisfaction with their well-being, school performance and life overall, although family satisfaction did not differ by sex. On the other hand, parents reported boys to have higher values regarding the CSHQ subdomains of sleep anxiety and night-time wakings. There were no gender disparities in the other CSHQ subdomains, the other objective or subjective sleep measurements, or self-reported functioning and anxiety. Notably, children reporting feelings of worry, sadness, or unhappiness exhibited reduced overall satisfaction and more sleep problems, as reported by both the child and their parents.

Earlier research has highlighted sex differences in ADHD symptom subtypes, with girls more often displaying inattention rather than impulsivity [12] and hyperactivity [12,60]. Our study corroborated this for inattention but not for hyperactivity. Historically, ADHD research has primarily focused on males [9], with limited representations of girls. Moreover, earlier ADHD studies have predominately relied on subjective measures, making sex differences in ADHD less clear [61]. Therefore, more studies examining ADHD presentations in girls are essential to confirm, extend, or contradict our findings and earlier findings [11].

Regarding the CORS items, girls in our cohort reported significantly lower values in well-being, school, and life overall, as well as in the total score, suggesting that girls with un-complicated ADHD and sleep problems may be in need of more support than boys with the same diagnosis. The results for the girls regarding satisfaction with functioning in school were particularly low, which perhaps prompts a need for special efforts in this group concerning school. It would be interesting to have these findings of sex differences replicated in other ADHD sample populations regarding the occurrence and the possible causes of these sex differences.

We measured sleep using both objective and subjective measures. In our cohort, no significant sex differences emerged in the objective measures conducted using the ActiGraph measurements. When comparing TST in our cohort to a study with 70 Danish children with ADHD aged 6–13 years, we found similar TST values [62]. However, in comparison to a study of 26 typical children, our cohort exhibited lower TST values in polysomnographic measurements [63]. These results align with a number of earlier studies which show that children with ADHD tend to have shorter sleep times than their healthy peers [64]. Interestingly, while one systematic review showed that children above nine years had longer TSTs than younger children [64]; in our cohort, those younger than nine years had longer sleep times than their older counterparts (see Supplementary Materials). A randomised controlled trial (RCT) of the same group of children found that weighted blankets significantly improved ActiGraph-measured WASO, TST, and SE, but not SOL, compared to using a lighter control blanket [44].

We used parent-reported CSHQ and child-reported ISI values for subjective measurements of sleep and found statistically significant sex differences regarding sleep anxiety and night-time wakings, where the boys had higher values. Regarding the other CSHQ subdomains and the total score, there were no gender disparities. According to the total CSHQ sums, parents reported sleep problems above the clinical cut-off of 41. When comparing the results from CSHQ in our cohort with other studies using CSHQ in children with ADHD, we found studies with both lower and higher values regarding total scores [65,66,67]. As mentioned above, our cohort and the cohorts we have compared our results with both reported values above the clinical cut-off for sleep problems, which agrees with earlier studies which conclude that parents of children with ADHD often report sleep problems in their children [14,34,36]. However, regarding the severity of the sleep problems, our cohort of children showed lower values than children with moderate or severe sleep problems, suggesting that uncomplicated ADHD, compared to typical clinical cases of ADHD, is associated with milder sleep disturbances, at least at a group level.

ISI explores child-reported issues with insomnia, whereas the parent-rated CSHQ focuses on more aspects of sleep problems, so the measures are hard to compare. However, at a group level, the children did not report any problems with insomnia, and their values indicated sub-threshold insomnia ranging from 8 to 14. In earlier research, it was shown that insomnia-related problems were associated with more severe forms of ADHD [68], and since the children in our cohort had uncomplicated ADHD, this may be one reason for the lack of self-reported sleep problems in our cohort. It was interesting, however, that parents reported sleep problems while the children did not experience problems with falling asleep. It has been shown that children with ADHD of different ages have different kinds of sleep problems [21,69]. Regarding insomnia, older children with autism spectrum disorder have more often reported problems with insomnia than younger children [70]. Maybe the relatively young age of the children in our cohort explains their lack of self-experienced problems with insomnia. The RCT on the same group of children showed that weighted blankets decreased sleep problems, causing the total scores of CSHQ and ISI to range within normative values for children with ADHD [44].

Regarding self-reported anxiety in our cohort, we did not find any differences between the sexes and found no studies with children with ADHD and sleep problems to compare our results. However, in a study of 42 typically developing children aged between 3 and 9 years old between, before, and after day surgery, and using a version with pictures instead of numbers, it was shown that our cohort had higher mean values than the values those children reported both before and after surgery [59].

Regarding functioning in daily life, the EQ-5D-Y questionnaires showed no differences between the sexes, and the majority of the children responded that they had no problems with the different items. The item that most children reported problems for was the one focusing on feeling worried, sad, or unhappy, which may depend on the ADHD diagnosis and the sleep problems. When comparing the EQ-5D-Y results in our sample with those of 307 children with ADHD from UK aged between 10 and 15 years, we found that they graded their overall health very similarly to the boys and girls in our cohort [71].

In our last aim, we wanted to examine whether there were any differences between children who reported problems such as worry, sadness, or unhappiness in the EQ-5D-Y compared to children who did not report these issues. We found statistically significant differences between these two groups regarding four of the questionnaires—CSHQ, ISI, and short STAI—all regarding total scores, as well as for four of the CORS items, where the group that felt worried, sad, or unhappy also had higher values of total scores in the CSHQ, ISI, and short STAI, as well as lower values on all CORS items. We did not find any other studies that had investigated this EQ-5D-item alone in relation to children with ADHD and sleep problems, but our results are in line with other studies that show an overlap between anxiety, depression, and disturbed sleep [43]. Our results indicate that children with uncomplicated ADHD and sleep problems who reported feeling worried, sad, or unhappy may have more sleep problems and may, therefore, need more support than children with only minor problems.

This study using baseline data from children with uncomplicated ADHD and sleep problems is based on a consecutive cohort of children with uncomplicated ADHD and sleep problems, where the sex distribution indicates a recruitment that corresponds to the epidemiological occurrence of ADHD. Another strength is the relatively high number of girls in this cohort of children. The study is subject to some limitations, as there was no control group included in this study, and our results have therefore been compared to other studies with children of the same age with or those without ADHD. Generalizability to standard clinical ADHD could be hampered both by excluding comorbid cases with severe social stress and also by the fact that more than half of the parents had a university degree, probably owing to the selection ahead of participating in an RCT that would be somewhat demanding on parents. We made multiple independent comparisons, which increased the risk of false positive results, especially for *p*-values above 0.01. In addition to that, it was difficult to compare studies due to the use of disparate rating scales and outcome measures. We had to use the ISI version for adolescents since it is not otherwise validated in populations with young children. However, it was important to let the children report if they had problems regarding these areas, and we used a version with simplified language to add clarity for the younger children.

## 5. Conclusions

Boys with uncomplicated ADHD and sleep problems were reported to have more problems with sleep anxiety and night-time wakings, indicating that they may need more support from the healthcare system regarding these specific sleep-related problems. Girls reported less satisfaction with functioning in daily life, well-being, school, and life overall, which indicates that girls with uncomplicated ADHD and sleep problems may need extra support from the healthcare system. Both boys and girls who experienced worry, sadness, or unhappiness reported worse sleep problems and were also less satisfied with their lives, which indicates more need for support if such symptoms are reported.

## Figures and Tables

**Table 1:** Characteristics of the study sample divided by sex.

Variables	Boys (*n* = 55)	Girls (*n* = 41)	*p*
**Age**			
Median	9.00	10.00 ^	**0.011**
Range	6–13	6–14	
**Diagnosis**			
Attention deficit, *n* (%)	10 (18)	15 (37) ”	**0.023**
Hyperactivity, *n* (%)	3 (6)	0 (0)	
Combined hyperactivity/attention deficit, *n* (%)	42 (76)	26 (63)	
**Medication**			
Melatonin, *n* (%)	6 (10.8)	3 (7.8) ”	0.449
Stimulants, *n* (%)	31 (55)	16 (40) *	0.103
**Iso Body mass index**			
Underweight, *n* (%)	2 (3.6)	3 (7.3) ”	0.814
Normal weight, *n* (%)	32 (57.1)	23 (58)	
Overweight, *n* (%)	14 (25.5)	8 (19.5)	
Obesity, *n* (%)	8 (14.5)	6 (14.6)	
**Parental education**			
University, *n* (%)	32 (58.2)	23 (56) ”	1.000
Upper secondary school, *n* (%)	20 (36.3)	15 (36.5)	
Elementary school, *n* (%)	3 (5.4)	3 (7.3)	
**Parental age**			
20–30, *n* (%)	2 (3.6)	1 (2.4) ”	0.694
31–40, *n* (%)	36 (65.5)	21 (51.2)	
41–50, *n* (%)	14 (25.5)	16 (39)	
>50, *n* (%)	3 (5.4)	3 (7.3)	

^^^ Differences in age between the sexes were examined with the Mann–Whitney U test. Differences between the sexes regarding diagnoses, medication, BMI, parental education, and parental age were examined with * Chi-squared test or ” Fisher’s exact test. A *p*-value < 0.05 was considered to be statistically significant. Bold numbers represent statistically significant values. Regarding diagnoses, the children diagnosed with hyperactivity were analysed together with the children with a combination of hyperactivity and attention deficit.

**Table 2:** ActiGraph measurements divided by sex.

ActiGraph	Boys (*n* = 55)	Girls (*n* = 41)	*p*
**Number of days (*n*)**			
Mean, Sd, Median	6.85 ± 0.41, 7.00	6.68 ± 0.66, 7.00	0.188
7	47	31	
6	6	5	
5	1	4	
Missing (*n*)	1	1	
**Total sleep time** (mean, sd, median)	493.37 ± 43.57, 489.07	482.94 ± 59.70, 475.57	0.468
**Sleep onset latency** (mean, sd, median)	36.04 ± 25.04, 29.86	32.76 ± 32.71, 21.00	0.175
**Sleep efficiency** (mean, sd, median)	86.26 ± 4.16, 86.61	86.99 ± 5.41, 88.22	0.206
**Wake after sleep onset** (mean, sd, median)	43.02 ± 14.69, 39.36	40.26 ± 18.92, 38.71	0.206

Mann–Whitney U tests were used in all analyses. A *p*-value < 0.05 was considered to be statistically significant.

**Table 3:** Subjectively reported sleep, Child Sleep Habit Questionnaire, and Insomnia Severity Index.

Questionnaires	Boys (*n* = 55)	Girls (*n* = 41)	*p*
**CSHQ (parent-reported)**			
Bedtime resistance (mean, sd, median)	9.64 ± 3.36, 9.00	8.85 ± 3.02, 8.00	0.324
Missing (*n*)	0	0	
Sleep onset delay (mean, sd, median)	2.27 ± 0.80, 2.00	2.32 ± 0.61, 2.00	0.965
Missing (*n*)	0	0	
Sleep duration (mean, sd, median)	5.27 ± 1.75, 5.00	5.63 ± 1.66, 6.00	0.322
Missing (*n*)	0	0	
Sleep anxiety (mean, sd, median)	6.73 ± 2.54, 7.00	5.63 ± 2.35, 5.00	**0.022**
Missing (*n*)	2	1	
Night-time awakenings (mean, sd, median)	5.04 ± 1.86, 5.00	4.15 ± 1.67, 4.00	**0.032**
Missing (*n*)	0	1	
Parasomnias (mean, sd, median)	9.13 ± 1.92, 9.00	8.44 ± 2.48, 8.00	0.073
Missing (*n*)	0	1	
Sleep disorder breathing (mean, sd, median)	3.20 ± 0.45, 3.00	3.10 ± 0.74, 3.00	0.794
Missing (*n*)	1	2	
Daytime sleepiness (mean, sd, median)	15.15 ± 3.79, 15:00 *	16.10 ± 3.14, 16.00	0.097
Missing (*n*)	1	0	
Total CSHQ score (mean, sd, median)	52.87 ± 7.71, 53.00	51.32 ± 7.88, 51.00	0.458
**ISI (child-reported)**			
Total score (mean, sd, median)	9.55 ± 5.01, 9.00	10.37 ± 4.90, 10.00	0.280

Mann–Whitney U tests were used in all the analyses except the one marked by an asterisk, wherein Student’s *t*-test was used. CSHQ range, 33–99; scores above 41 indicate sleep disturbances. ISI range, 0–28; values between 15 and 21 indicate clinical insomnia and those between 22 and 28 indicate severe insomnia. A *p*-value < 0.05 was considered to be statistically significant. Bold numbers represent statistically significant values. Child Sleep Habit Questionnaire, CSHQ: Insomnia Severity Index, ISI.

**Table 4:** The parent-reported Swanson, Nolan and Pelham Scale and the short form of the State-Trait Anxiety Inventory divided by sex.

Measure Instrument	Boys (*n* = 55)	Girls (*n* = 41)	*p*
**SNAP IV (parent reported)**			
Inattention score, (mean, sd)	16.87 ± 5.33	18.71 ± 4.51 *	**0.039**
Hyperactivity/impulsivity score (mean, sd)	14.82 ± 5.67	15.39 ± 6.07 *	0.318
Inattention			
(*n* without missing answers)	53	40	
(*n* with <3 missing answers)	3	1	
<13	13	4	
13–17	17	13	
18–22	17	13	
23–27	8	11	
Missing (*n*)	0	0	
Hyperactivity/impulsivity			
(*n* without missing answers)	49	38	
(*n* with <4 missing answers)	7	3	
<13	18	10	
13–17	18	15	
18–22	11	11	
23–27	8	5	
Missing (*n*)	0	0	
**Short STAI (child-reported)**			
Total score (mean, sd, median)	10.64 ± 3.37, 11.00	11.44 ± 4.02, 10.00 ^	0.420
Range	6–19	6–19	

* Student’s *t*-tests were used. ^ Mann–Whitney U test was used. In clinical practice, the following cut-offs are used for results from SNAP: <13, no inattention or hyperactivity; 13–17, mild; 18–22 moderate; and 23–27, severe. A *p*-value < 0.05 was considered to be statistically significant. Bold numbers represent statistically significant values. Swanson, Nolan and Pelham rating scale IV, SNAP IV-parent; short State-Trait Anxiety Inventory, short STAI.

**Table 5:** EuroQoL-5 dimension questionnaire and Child Outcome Rating scale divided by sex.

Self-Reported Questionnaires		Boys (*n* = 55)	Girls (*n* = 41)	*p*
**EQ-5D-Y (child-reported)**				
Mobility	No problems with mobility	56 (100%)	39 (95%) ”	0.180
	Some/a lot of problems with mobility	0 (0%)	2 (5%)	
Looking after myself	No problems looking after myself	35 (64%)	30 (73%) *	0.381
	Some/a lot of problems looking after myself	20 (36%)	11 (27%)	
Doing usual activities	No problems doing usual activities	37 (67%)	31 (76%) *	0.497
	Some/a lot of problems doing usual activities	18 (33%)	10 (24%)	
Having pain or discomfort	No pain or discomfort	44 (80%)	26 (63%) *	0.103
	Some/a lot of pain and discomfort	11 (20%)	15 (37%)	
Feeling worried sad or unhappy	Not worried, sad, or unhappy	30 (54%)	20 (49%) *	0.680
	A bit worried, sad, or unhappy	25 (46%)	21 (51%)	
**EQ-5D-Y, VAS (child-reported)** (mean, sd, median)		82.42 ± 19.18, 86.00	80.39 ± 16.61, 80.00	0.417
**CORS (child-reported)**				
Total score, (mean, sd, median)		32.59 ± 6.36, 34.00	29.29 ± 5.94, 29.40 ^	**0.006**
≥28 (*n*)		46	24	
<28 (*n*)		9	17	
Well-being (mean, sd median)		8.54 ± 2.04, 9.90	7.41 ± 1.91, 7.80 ^	**0.001**
Family (mean, sd, median)		8.32 ± 1.95, 9.00	8.54 ± 1.79, 9.10 ^	0.649
School (mean, sd, median)		7.39 ± 2.63, 8.00	5.94 ± 2.52, 6.00 ^	**0.004**
Life overall (mean, sd, median)		8.34 ± 2.09, 9.10	7.39 ± 2.37, 8.10 ^	**0.017**

EQ-5D-Y, ” Fisher’s exact test or * Chi-squared tests were used to examine differences between the sexes. EuroQol-5 Dimensions-Youth, EQ-5D-Y; Child Outcome Rating scale, CORS, ^ Mann–Whitney U tests were used to examine differences between the sexes. A *p*-value < 0.05 was considered to be statistically significant. Bold numbers represent statistically significant values.

**Table 6:** Subjectively reported sleep, anxiety, and satisfaction with functioning in daily life divided by children who reported feeling worried, sad, or unhappy or not in the EQ-5D-Y questionnaire.

Questionnaires	Not Worried, Sad, or Unhappy (*n* = 50)	Worried, Sad, or Unhappy (*n* = 46)	*p*
**CSHQ**			
Total sum (mean, sd, median)	50.64 ± 7.66, 50.50	53.91 ± 7.63, 55.00	**0.027**
Missing (*n*)	2	1	
**ISI**			
Total score (mean, sd, median)	8.14 ± 4.78, 7.00	11.80± 4.44, 10.50	**<0.001**
**Short STAI**			
Total score (mean, sd, median)	9.78 ± 3.48, 9.00	12.28 ± 3.43, 12.00	**<0.001**
Range	6–19	6–19	
**CORS**			
Total score, (mean, sd, median)	34.13 ± 5.12, 35.25	27.98 ± 6.07, 28.60	**<0.001**
≥28 (*n*)	45	25	
<28 (*n*)	5	21	
Well-being (mean, sd median)	8.92 ± 1.43, 9.80	7.12 ± 2.23, 7.40	**0.013**
Family (mean, sd, median)	8.90 ± 21.60, 9.80	7.89 ± 2.03, 8.55	**0.030**
School (mean, sd, median)	7.32± 2.48, 7.75	6.17 ± 2.76, 6.00	**<0.001**
Life overall (mean, sd, median)	8.99 ± 1.34, 9.85	6.79 ± 2.49, 7.25	**<0.001**

Mann–Whitney U tests were used. CSHQ range, 33–99; scores above 41 indicate sleep disturbances. ISI range, 0–28, values between 15 and 21 indicate clinical insomnia and between 22 and 28 indicate severe insomnia. CORS, item range 0–10; total score range 0–40. A *p*-value < 0.05 was considered to be statistically significant. Bold numbers represent statistically significant values. Child Sleep Habit Questionnaire, CSHQ: Insomnia Severity Index, ISI; short form of the State-Trait Anxiety Inventory, short STAI; Child Outcome Rating Scale, CORS.

## Data Availability

Datasets are available through the corresponding author upon reasonable request.

## Appendix A

**Table A1:** Characteristics of the study sample divided by children who reported feeling worried, sad, or unhappy or who did not report that in the EQ-5D-Y questionnaire.

Variables	Not Worried, Sad, or Unhappy (*n* = 50)	Worried, Sad, or Unhappy (*n* = 46)	*p*
**Age**			
Median	10.00	9.00 ^	0.232
Range	6–14	6–13	
**Diagnosis**			
Attention deficit, *n* (%)	13 (26)	12 (26) ”	0.992
Hyperactivity, *n* (%)	1 (2)	2 (4)	
Combined hyperactivity and attention deficit, *n* (%)	36 (72)	32 (70)	
**Medication**			
Melatonin, *n* (%)	3 (6)	6 (13) ”	0.163
Stimulants, *n* (%)	25 (50)	22 (47.8) *	0.588
**Iso Body mass index**			
Underweight, *n* (%)	4 (8)	1 (2) ”	0.136
Normal weight, *n* (%)	28 (56)	27 (58.7)	
Overweight, *n* (%)	14 (28)	8 (17.3)	
Obesity, *n* (%)	4 (8)	10 (21.7)	
**Parental education**			
University, *n* (%)	26 (52.1)	29 (63) ”	0.244
Upper secondary school, *n* (%)	22 (44)	13 (28.3)	
Elementary school, *n* (%)	2 (4)	4 (8.7)	
**Parental age**			
20–30, *n* (%)	2 (4)	1 (2.1) ”	0.786
31–40, *n* (%)	28 (56)	29 (63)	
41–50, *n* (%)	15 (30)	15 (32)	
>50, *n* (%)	5 (10)	1 (2.1)	

^ Differences in age between the sexes have been examined with the Mann–Whitney U test. Differences in diagnoses between the sexes have been examined with * Chi-squared test or ” Fisher’s exact tests. A *p*-value < 0.05 was considered to be statistically significant. Regarding diagnosis, the children diagnosed with hyperactivity were analysed together with the children with a combination of hyperactivity and attention deficit. *p*-value < 0.05 was considered to be statistically significant.

**Table A2:** ActiGraph measurements divided by children who reported feeling worried, sad, or un- happy or who did not report that in the EQ-5D-Y questionnaire.

ActiGraph	Not Worried, Sad, or Unhappy (*n* = 50)	Worried, Sad, or Unhappy (*n* = 46)	*p*
**Number of days (*n*)**			
Mean, Sd, Median	6.71 ± 0.61, 7.00	7.00	0.972
7	39	39	
6	6	5	
5	4	1	
Missing (*n*)	1	1	
**Total sleep time** (mean, sd, median)	481.37 ± 53.98, 470.29	497.18 ± 46.79, 500.29	0.134
**Sleep onset latency** (mean, sd, median)	38.30 ± 34.63, 27.20	30.66 ± 19.24, 27.29	0.384
**Sleep efficiency** (mean, sd, median)	86.29 ± 4.13, 86.63	87.16 ± 3.61, 87.67	0.771
**Wake after sleep onset** (mean, sd, median)	41.40 ± 16.86, 39.43	42.32 ± 16.46, 40.00	0.438

Mann–Whitney U tests were used. A *p*-value < 0.05 was considered to be statistically significant.

**Table A3:** Subjectively reported sleep in Child Sleep Habit Questionnaire, parent, and Insomnia Severity Index, divided by children who reported feeling worried, sad, or unhappy or who did not report that in the EQ-5D-Y questionnaire.

Questionnaires	Not Worried, Sad, or Unhappy (*n* = 50)	Worried, Sad, or Unhappy (*n* = 46)	*p*
**CSHQ**			
Total sum (mean, sd, median)	50.64 ± 7.66, 50.50	53.91 ± 7.63, 55.00	**0.027**
Missing (*n*)	2	1	
**ISI**			
Total score (mean, sd, median)	8.14 ± 4.78, 7.00	11.80 ± 4.44, 10.50	**<0.001**

Mann–Whitney U tests were used. CSHQ range, 33–99, scores above 41 indicates sleep disturbances. ISI range, 0–28, values between 15 and 21 indicate clinical insomnia and between 22 and 28 indicate severe insomnia. A *p*-value < 0.05 was considered statistically significant. Bold numbers represent statistically significant values. Child Sleep Habit Questionnaire, CSHQ: Insomnia Severity Index, ISI; short form of the State-Trait Anxiety Inventory. These analyses are also included in the manuscript.

**Table A4:** The parent-reported Swanson Nolan and Pelham rating scale and the short form of the State-Trait Anxiety Inventory divided by children who reported feeling worried, sad, or unhappy or who did not report that in the EQ-5D-Y questionnaire.

Measure Instrument	Not Worried, Sad, or Unhappy (*n* = 50)	Worried, Sad, or Unhappy (*n* = 46)	*p*
**SNAP IV-parent**			
Inattention score, (mean, sd)	18.12 ± 4.91	17.15 ± 5.22 *	0.351
Hyperactivity/impulsivity score (mean, sd)	14.78 ± 5.50	15.37 ± 6.20 *	0.623
Inattention			
<13	8	9	
13–17	15	15	
18–22	15	25	
23–27	12	7	
Missing (*n*)	0	0	
Hyperactivity/impulsivity			
<13	16	12	
13–17	17	16	
18–22	11	11	
23–27	6	7	
Missing (*n*)	0	0	
**Short STAI**			
Total score	9.78 ± 3.48, 9.00	12.28 ± 3.43, 12.00 ^	**<0.001**
Range	6–19	6–19	

* Student’s *t*-tests were used. ^ Mann–Whitney U test was used. In clinical practice, the following cut-offs are used for results from SNAP: <13, no inattention or hyperactivity, 13–17, mild, 18–22 moderate and 23–27, severe. *p*-value < 0.05 was considered statistically significant. Bold numbers represent statistically significant values. Swanson, Nolan and Pelham scale IV, SNAP IV-parent; short State-Trait Anxiety Inventory, Short STAI. The short STAI-analyses are also included in the manuscript.

**Table A5:** Child Outcome Rating Scale divided by children who reported feeling worried, sad, or unhappy or those who did not report that in the EQ-5D-Y questionnaire.

Questionnaire	Not Worried, Sad, or Unhappy	Worried, Sad, or Unhappy	*p*
	(*n* = 50)	(*n* = 46)	
**CORS**			
total score (mean, sd, median)	34.13 ± 5.12, 35.25	27.98 ± 6.07, 28.60	**<0.001**
≥28 (*n*)	45	25	
<28 (*n*)	5	21	
Me (mean, sd, median)	8.92 ± 1.43, 9.80	7.12 ± 2.23, 7.40	**0.013**
Family (mean, sd, median)	8.90 ± 21.60, 9.80	7.89 ± 2.03, 8.55	**0.030**
School (mean, sd, median)	7.32 ± 2.48, 7.75	6.17 ± 2.76, 6.00	**<0.001**
Life (mean, sd, median)	8.99 ± 1.34, 9.85	6.79 ± 2.49, 7.25	**<0.001**

Mann–Whitney U tests were used. CORS, item range 0–10, total score range 0–40. A *p*-value < 0.05 was considered statistically significant. Bold numbers represent statistically significant values. Child Outcome Rating Scale, CORS. These analyses are also included in the manuscript.

**Table A6:** Comparison between total sleep time in children younger than 9 years and children older than 9 years with Student’s *t*-test.

Total Sleep Time	<9 Years of Age	>9 Years of Age	*p*
	(*n* = 37)	(*n* = 57)	
Mean, sd	521 ± 40.77	467.78 ± 45.79	**<0.001**
Missing (*n*)	0	2	

The bold number respresent a statistically significant value.

## References

[1] Salari N., Ghasemi H., Abdoli N., Rahmani A., Shiri M.H., Hashemian A.H., Akbari H., Mohammadi M. (2023). The global prevalence of ADHD in children and adolescents: A systematic review and meta-analysis. Ital. J. Pediatr..

[2] (2013). Diagnostic and Statistical Manual of Mental Disorders (DSM-5) [Internet].

[3] Russell G., Ford T., Rosenberg R., Kelly S. (2014). The association of attention deficit hyperactivity disorder with socioeconomic disadvantage: Alternative explanations and evidence. J. Child Psychol. Psychiatry.

[4] Biederman J., Faraone S.V. (2005). Attention-deficit hyperactivity disorder. Lancet.

[5] Faraone S.V., Banaschewski T., Coghill D., Zheng Y., Biederman J., Bellgrove M.A., Newcorn J.H., Gignac M., Al Saud N.M., Manor I. (2021). The World Federation of ADHD International Consensus Statement: 208 Evidence-based conclusions about the disorder. Neurosci. Biobehav. Rev..

[6] The Social Welfare Board Diagnoses in Inpatient Care and Specialised Outpatient Care. https://www.socialstyrelsen.se/statistik-och-data/statistik/statistikdatabasen.

[7] Gershon J. (2002). A meta-analytic review of gender differences in ADHD. J. Atten. Disord..

[8] Hinshaw S.P., Nguyen P.T., O’Grady S.M., Rosenthal E.A. (2022). Annual Research Review: Attention-deficit/hyperactivity disorder in girls and women: Underrepresentation, longitudinal processes, and key directions. J. Child Psychol. Psychiatry.

[9] Young S., Adamo N., Asgeirsdottir B.B., Branney P., Beckett M., Colley W., Cubbin S., Deeley Q., Farrag E., Gudjonsson G. (2020). Females with ADHD: An expert consensus statement taking a lifespan approach providing guidance for the identification and treatment of attention-deficit/hyperactivity disorder in girls and women. BMC Psychiatry.

[10] Carucci S., Narducci C., Bazzoni M., Balia C., Donno F., Gagliano A., Zuddas A. (2023). Clinical characteristics, neuroimaging findings, and neuropsychological functioning in attention-deficit hyperactivity disorder: Sex differences. J. Neurosci. Res..

[11] Quinn P.O., Madhoo M. (2014). A review of attention-deficit/hyperactivity disorder in women and girls: Uncovering this hidden diagnosis. Prim. Care Companion CNS Disord..

[12] Rucklidge J.J. (2010). Gender differences in attention-deficit/hyperactivity disorder. Psychiatr. Clin. N. Am..

[13] Cortese S., Faraone S.V., Konofal E., Lecendreux M. (2009). Sleep in children with attention-deficit/hyperactivity disorder: Meta-analysis of subjective and objective studies. J. Am. Acad. Child Adolesc. Psychiatry.

[14] Singh K., Zimmerman A.W. (2015). Sleep in Autism Spectrum Disorder and Attention Deficit Hyperactivity Disorder. Semin. Pediatr. Neurol..

[15] Van Dongen H.P., Maislin G., Mullington J.M., Dinges D.F. (2003). The cumulative cost of additional wakefulness: Dose-response effects on neurobehavioral functions and sleep physiology from chronic sleep restriction and total sleep deprivation. Sleep.

[16] Gross D.W., Gotman J. (1999). Correlation of high-frequency oscillations with the sleep-wake cycle and cognitive activity in humans. Neuroscience.

[17] Gosling C.J., Cortese S., Konofal E., Lecendreux M., Faraone S.V. (2023). Association of Parent-Rated Sleep Disturbances With Attention-Deficit/Hyperactivity Disorder Symptoms: 9-Year Follow-up of a Population-Based Cohort Study. J. Am. Acad. Child Adolesc. Psychiatry.

[18] Bondopadhyay U., Diaz-Orueta U., Coogan A.N. (2022). A Systematic Review of Sleep and Circadian Rhythms in Children with Attention Deficit Hyperactivity Disorder. J. Atten. Disord..

[19] Lucas I., Mulraney M., Sciberras E. (2019). Sleep problems and daytime sleepiness in children with ADHD: Associations with social, emotional, and behavioral functioning at school, a cross-sectional study. Behav. Sleep Med..

[20] Sung V., Hiscock H., Sciberras E., Efron D. (2008). Sleep problems in children with attention-deficit/hyperactivity disorder: Prevalence and the effect on the child and family. Arch. Pediatr. Adolesc. Med..

[21] Martin C.A., Papadopoulos N., Chellew T., Rinehart N.J., Sciberras E. (2019). Associations between parenting stress, parent mental health and child sleep problems for children with ADHD and ASD: Systematic review. Res. Dev. Disabil..

[22] Chaput J.P., Gray C.E., Poitras V.J., Carson V., Gruber R., Olds T., Weiss S.K., Gorber S.C., Kho M.E., Sampson M. (2016). Systematic review of the relationships between sleep duration and health indicators in school-aged children and youth. Appl. Physiol. Nutr. Metab. Physiol. Appl. Nutr. Metabolisme.

[23] Schlieber M., Han J. (2021). The Role of Sleep in Young Children’s Development: A Review. J. Genet. Psychol..

[24] Corkum P., Tannock R., Moldofsky H., Hogg-Johnson S., Humphries T. (2001). Actigraphy and parental ratings of sleep in children with attention-deficit/hyperactivity disorder (ADHD). Sleep.

[25] Hodgkins P., Setyawan J., Mitra D., Davis K., Quintero J., Fridman M., Shaw M., Harpin V. (2013). Management of ADHD in children across Europe: Patient demographics, physician characteristics and treatment patterns. Eur. J. Pediatr..

[26] Owens J.A. (2005). The ADHD and sleep conundrum: A review. J. Dev. Behav. Pediatr. JDBP.

[27] Yürümez E., Kılıç B.G. (2016). Relationship Between Sleep Problems and Quality of Life in Children With ADHD. J. Atten. Disord..

[28] Efron D., Lycett K., Sciberras E. (2014). Use of sleep medication in children with ADHD. Sleep Med..

[29] Quach J., Hiscock H., Wake M. (2012). Sleep problems and mental health in primary school new entrants: Cross-sectional community-based study. J. Paediatr. Child Health.

[30] Becker S.P., Cusick C.N., Sidol C.A., Epstein J.N., Tamm L. (2018). The impact of comorbid mental health symptoms and sex on sleep functioning in children with ADHD. Eur. Child Adolesc. Psychiatry.

[31] Yoon S.Y., Jain U., Shapiro C. (2012). Sleep in attention-deficit/hyperactivity disorder in children and adults: Past, present, and future. Sleep Med. Rev..

[32] Kirov R., Kinkelbur J., Heipke S., Kostanecka-Endress T., Westhoff M., Cohrs S., Ruther E., Hajak G., Banaschewski T., Rothenberger A. (2004). Is there a specific polysomnographic sleep pattern in children with attention deficit/hyperactivity disorder?. Sleep Res..

[33] Miano S., Donfrancesco R., Bruni O., Ferri R., Galiffa S., Pagani J., Montemitro E., Kheirandish L., Gozal D., Villa M.P. (2006). NREM sleep instability is reduced in children with attention-deficit/hyperactivity disorder. Sleep.

[34] Ball J.D., Tiernan M., Janusz J., Furr A. (1997). Sleep patterns among children with attention-deficit hyperactivity disorder: A reexamination of parent perceptions. J. Pediatr. Psychol..

[35] Stein D., Pat-Horenczyk R., Blank S., Dagan Y., Barak Y., Gumpel T.P. (2002). Sleep disturbances in adolescents with symptoms of attention-deficit/hyperactivity disorder. J. Learn. Disabil..

[36] Chervin R.D., Dillon J.E., Bassetti C., Ganoczy D.A., Pituch K.J. (1997). Symptoms of sleep disorders, inattention, and hyperactivity in children. Sleep.

[37] Owens J.A., Maxim R., Nobile C., McGuinn M., Msall M. (2000). Parental and self-report of sleep in children with attention-deficit/hyperactivity disorder. Arch. Pediatr. Adolesc. Med..

[38] Li S., Jin X., Yan C., Wu S., Jiang F., Shen X. (2009). Sleep problems in chinese school-aged children with a parent-reported history of ADHD. J. Atten. Disord..

[39] Lim C.G., Ooi Y.P., Fung D.S., Mahendran R., Kaur A. (2008). Sleep disturbances in Singaporean children with attention deficit hyperactivity disorder. Ann. Acad. Med. Singap..

[40] Agostini A., Centofanti S. (2021). Normal Sleep in Children and Adolescence. Child. Adolesc. Psychiatr. Clin. N. Am..

[41] Stražišar B.G. (2021). Sleep Measurement in Children-Are We on the Right Track?. Sleep Med. Clin..

[42] Riley A.W. (2004). Evidence that school-age children can self-report on their health. Ambul. Pediatr..

[43] Chorney D.B., Detweiler M.F., Morris T.L., Kuhn B.R. (2008). The interplay of sleep disturbance, anxiety, and depression in children. J. Pediatr. Psychol..

[44] Lönn M., Svedberg P., Nygren J., Jarbin H., Aili K., Larsson I. (2024). The efficacy of weighted blankets for sleep in children with attention-deficit/hyperactivity disorder—A randomized controlled crossover trial. J. Sleep Res..

[45] Larsson I., Aili K., Nygren J.M., Johansson P., Jarbin H., Svedberg P. (2022). SLEEP: Intervention with weighted blankets for children with attention deficit hyperactivity disorder (ADHD) and sleep problems: Study protocol for a randomised control trial. BMJ Open.

[46] Wernersson R., Johansson J., Andersson M., Jarbin H. (2020). Evaluation of a new model for assessment and treatment of uncomplicated ADHD—Effect, patient satisfaction and costs. Nord. J. Psychiatry.

[47] Sadeh A. (2011). The role and validity of actigraphy in sleep medicine: An update. Sleep Med. Rev..

[48] Owens J.A., Spirito A., McGuinn M. (2000). The Children’s Sleep Habits Questionnaire (CSHQ): Psychometric properties of a survey instrument for school-aged children. Sleep.

[49] Owens J., Maxim R., McGuinn M., Nobile C., Msall M., Alario A. (1999). Television-viewing habits and sleep disturbance in school children. Pediatrics.

[50] Larsson I., Svedberg P., Nygren J.M., Malmborg S.J. (2024). Validity and reliability of the Swedish version of the Children’s sleep Habits Questionnaire (CSHQ-SWE). BMC Pediatrics.

[51] Bastien C.H., Vallières A., Morin C.M. (2001). Validation of the Insomnia Severity Index as an outcome measure for insomnia research. Sleep Med..

[52] Chung K.F., Kan K.K., Yeung W.F. (2011). Assessing insomnia in adolescents: Comparison of Insomnia Severity Index, Athens Insomnia Scale and Sleep Quality Index. Sleep Med..

[53] Hall C.L., Guo B., Valentine A.Z., Groom M.J., Daley D., Sayal K., Hollis C. (2020). The Validity of the SNAP-IV in Children Displaying ADHD Symptoms. Assessment.

[54] Swanson J.M., Kraemer H.C., Hinshaw S.P., Arnold L.E., Conners C.K., Abikoff H.B., Clevenger W., Davies M., Elliott G.R., Greenhill L.L. (2001). Clinical relevance of the primary findings of the MTA: Success rates based on severity of ADHD and ODD symptoms at the end of treatment. J. Am. Acad. Child Adolesc. Psychiatry.

[55] Ravens-Sieberer U., Wille N., Badia X., Bonsel G., Burström K., Cavrini G., Devlin N., Egmar A.-C., Gusi N., Herdman M. (2010). Feasibility, reliability, and validity of the EQ-5D-Y: Results from a multinational study. Qual. Life Res..

[56] Miller S., Duncan B.I., Brown J. (2003). The Outcome Rating Scale: A Preliminary Study of the Reliability, Validity and Feasibility of a Brief Visual Analog Measure. J. Brief Ther..

[57] Duncan B.L., Sparks Jacqueline A., Miller S.D., Bohanske R.T., Claud D.A. (2006). Giving youth a voice: A preliminary study of the reliability and validity of a brief outcome measure for children, adolescents, and caretakers. J. Brief Ther..

[58] Marteau T.M., Bekker H. (1992). The development of a six-item short-form of the state scale of the Spielberger State-Trait Anxiety Inventory (STAI). Br. J. Clin. Psychol..

[59] Nilsson S., Buchholz M., Thunberg G. (2012). Assessing Children’s Anxiety Using the Modified Short State-Trait Anxiety Inventory and Talking Mats: A Pilot Study. Nurs. Res. Pract..

[60] Gaub M., Carlson C.L. (1997). Gender differences in ADHD: A meta-analysis and critical review. J. Am. Acad. Child Adolesc. Psychiatry.

[61] Slobodin O., Davidovitch M. (2019). Gender Differences in Objective and Subjective Measures of ADHD Among Clinic-Referred Children. Front. Hum. Neurosci..

[62] Virring A., Lambek R., Thomsen P.H., Møller L.R., Jennum P.J. (2016). Disturbed sleep in attention-deficit hyperactivity disorder (ADHD) is not a question of psychiatric comorbidity or ADHD presentation. J. Sleep Res..

[63] Godino J.G., Wing D., de Zambotti M., Baker F.C., Bagot K., Inkelis S., Pautz C., Higgins M., Nichols J., Brumback T. (2020). Performance of a commercial multi-sensor wearable (Fitbit Charge HR) in measuring physical activity and sleep in healthy children. PLoS ONE.

[64] Sadeh A., Pergamin L., Bar-Haim Y. (2006). Sleep in children with attention-deficit hyperactivity disorder: A meta-analysis of polysomnographic studies. Sleep. Med. Rev..

[65] Virring A., Lambek R., Jennum P.J., Møller L.R., Thomsen P.H. (2017). Sleep Problems and Daily Functioning in Children With ADHD: An Investigation of the Role of Impairment, ADHD Presentations, and Psychiatric Comorbidity. J. Atten. Disord..

[66] Chiraphadhanakul K., Jaimchariyatam N., Pruksananonda C., Chonchaiya W. (2016). Increased Sleep Disturbances in Thai Children With Attention-Deficit Hyperactivity Disorder Compared with Typically Developing Children. Behav. Sleep Med..

[67] Lycett K., Mensah F.K., Hiscock H., Sciberras E. (2015). Comparing subjective measures of behavioral sleep problems in children with ADHD: A cross-sectional study. Sleep Med..

[68] Li X., Shea K.S.C., Chiu W.V., Lau F.L.F., Wong C.K.D., Yu W.M.M., Li A.M., Wing Y.K., Lai Y.C.K., Li S.X. (2022). The associations of insomnia symptoms with daytime behavior and cognitive functioning in children with attention-deficit/hyperactivity disorder. J. Clin. Sleep Med. JCSM Off. Publ. Am. Acad. Sleep Med..

[69] Lunsford-Avery J.R., Krystal A.D., Kollins S.H. (2016). Sleep disturbances in adolescents with ADHD: A systematic review and framework for future research. Clin. Psychol. Rev..

[70] Goldman S.E., Richdale A.L., Clemons T., Malow B.A. (2012). Parental sleep concerns in autism spectrum disorders: Variations from childhood to adolescence. J. Autism Dev. Disord..

[71] Peasgood T., Bhardwaj A., Biggs K., Brazier J.E., Coghill D., Cooper C.L., Daley D., De Silva C., Harpin V., Hodgkins P. (2016). The impact of ADHD on the health and well-being of ADHD children and their siblings. Eur. Child Adolesc. Psychiatry.

